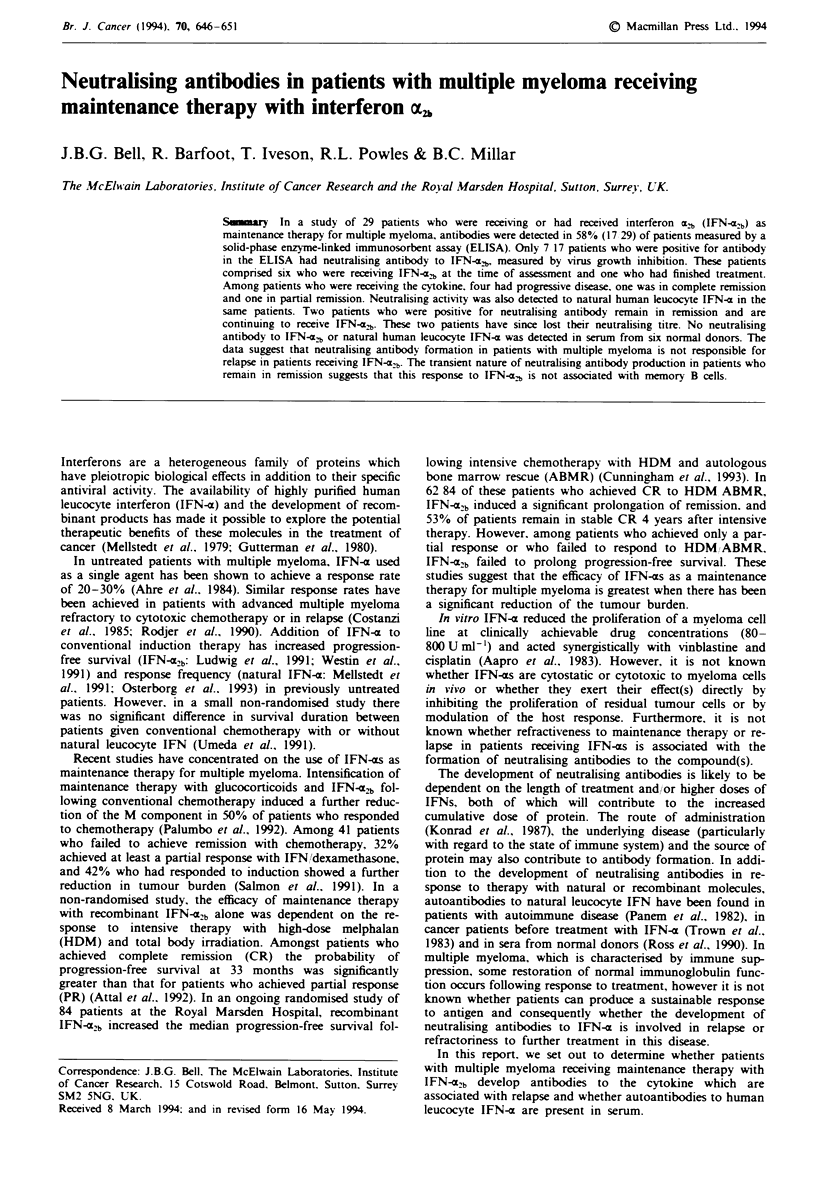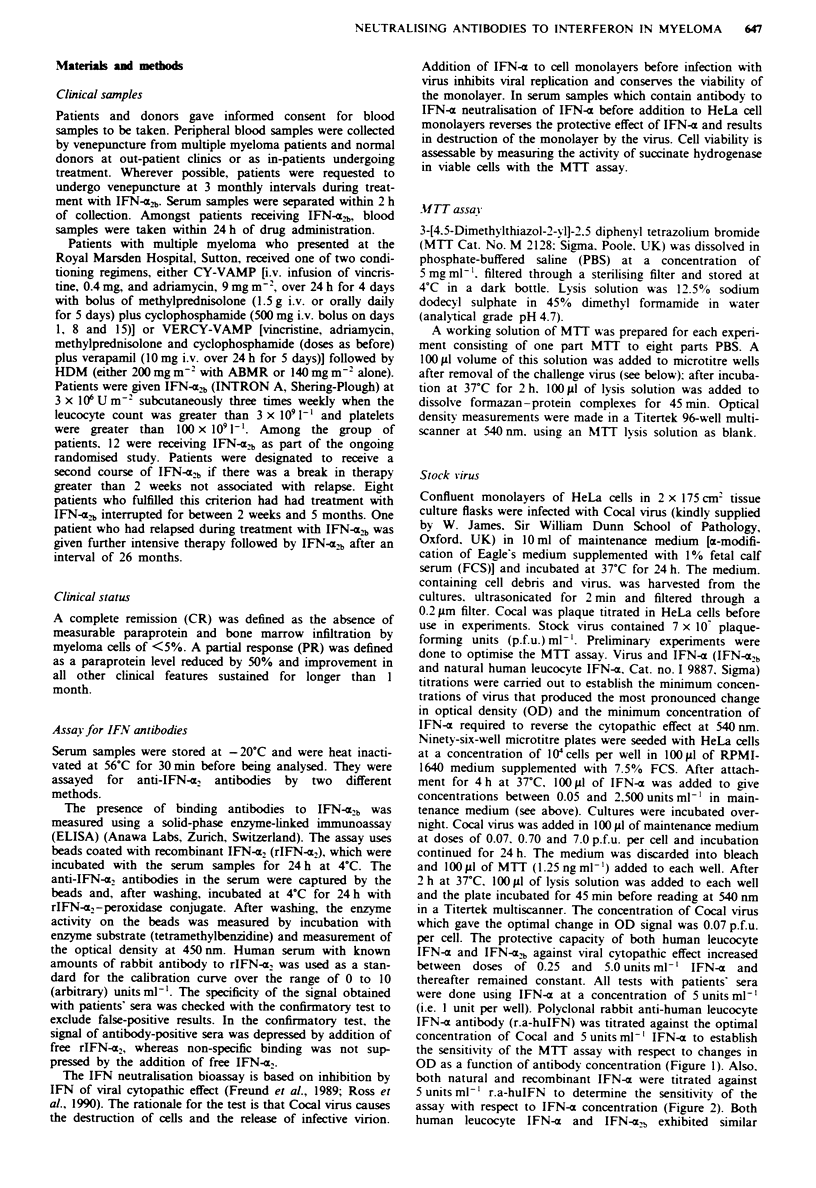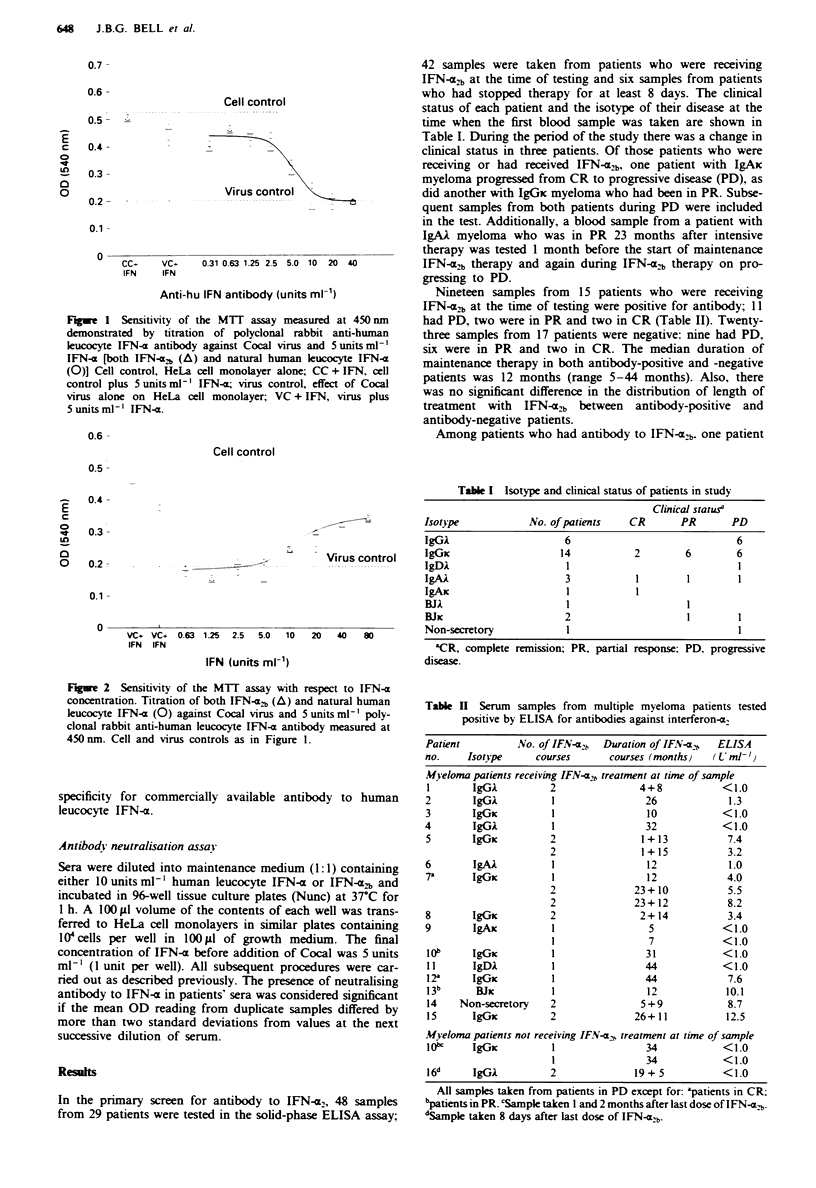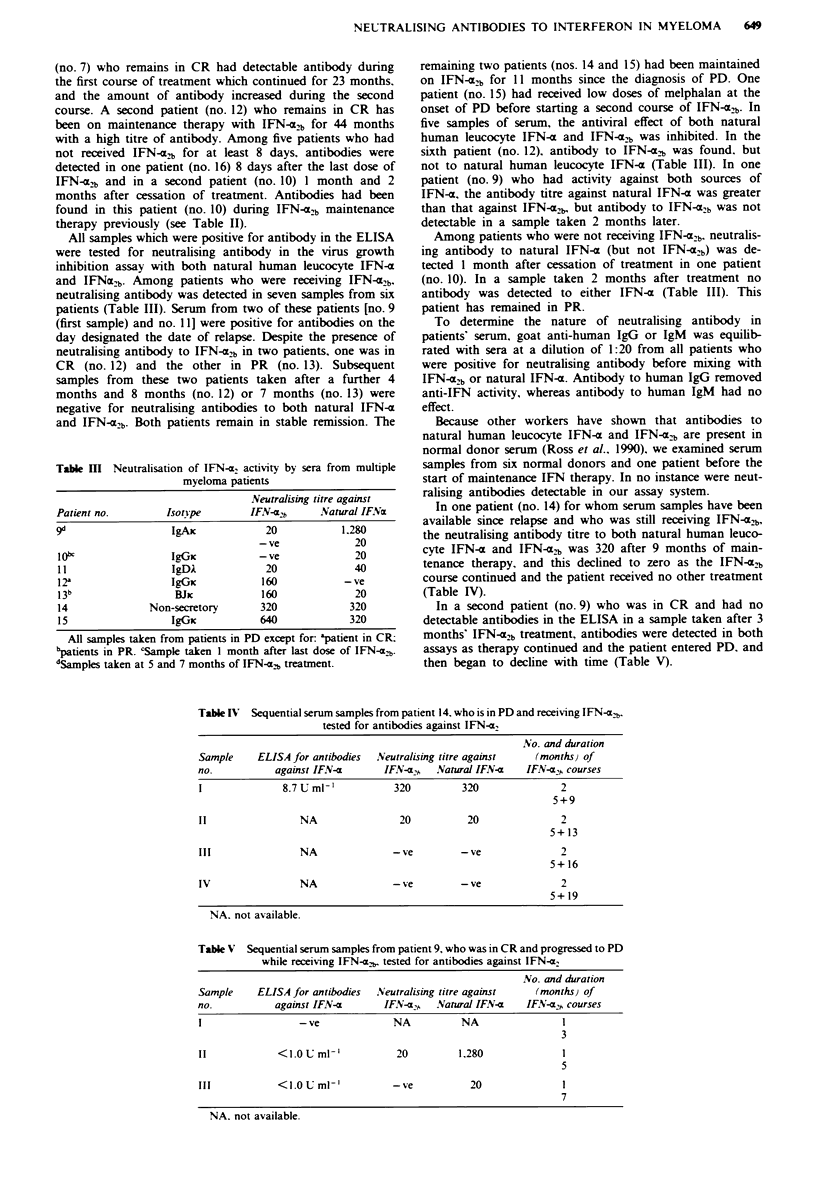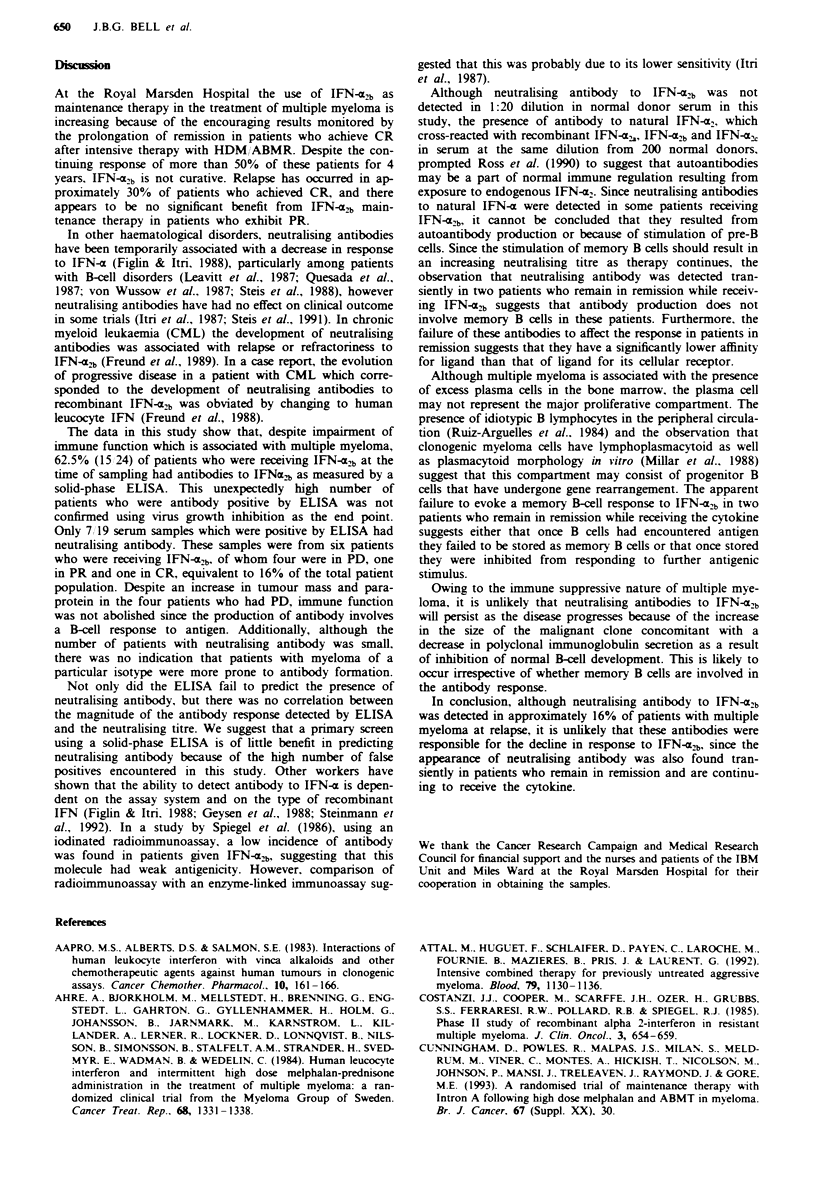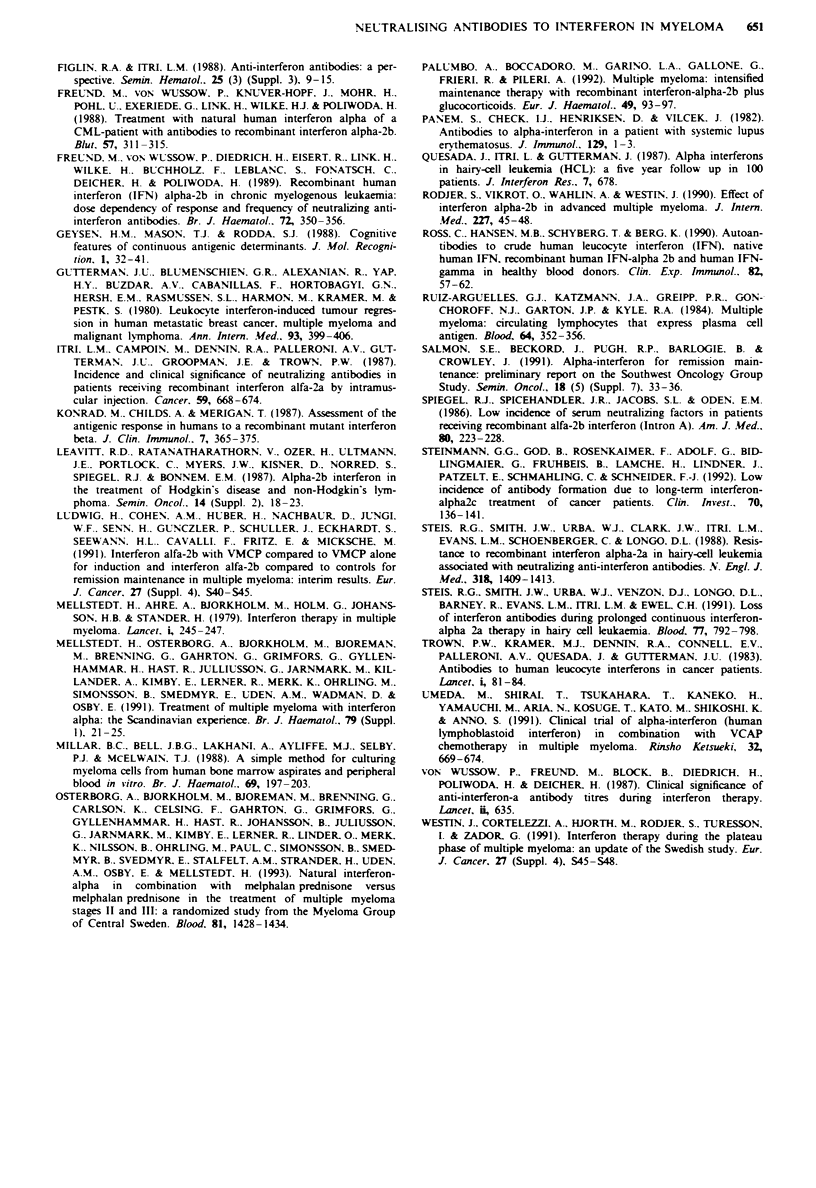# Neutralising antibodies in patients with multiple myeloma receiving maintenance therapy with interferon alpha 2b.

**DOI:** 10.1038/bjc.1994.365

**Published:** 1994-10

**Authors:** J. B. Bell, R. Barfoot, T. Iveson, R. L. Powles, B. C. Millar

**Affiliations:** McElwain Laboratories, Institute of Cancer Research, Sutton, Surrey, UK.

## Abstract

In a study of 29 patients who were receiving or had received interferon alpha 2b (IFN-alpha 2b) as maintenance therapy for multiple myeloma, antibodies were detected in 58% (17/29) of patients measured by a solid-phase enzyme-linked immunosorbent assay (ELISA). Only 7/17 patients who were positive for antibody in the ELISA had neutralising antibody to IFN-alpha 2b, measured by virus growth inhibition. These patients comprised six who were receiving IFN-alpha 2b at the time of assessment and one who had finished treatment. Among patients who were receiving the cytokine, four had progressive disease, one was in complete remission and one in partial remission. Neutralising activity was also detected to natural human leucocyte IFN-alpha in the same patients. Two patients who were positive for neutralising antibody remain in remission and are continuing to receive IFN-alpha 2b. These two patients have since lost their neutralising titre. No neutralising antibody to IFN-alpha 2b or natural human leucocyte IFN-alpha was detected in serum from six normal donors. The data suggest that neutralising antibody formation in patients with multiple myeloma is not responsible for relapse in patients receiving IFN-alpha 2b. The transient nature of neutralising antibody production in patients who remain in remission suggests that this response to IFN-alpha 2b is not associated with memory B cells.


Br. J. Cancer (1994), 70, 646-651 © Macmillan Press Ltd., 1994

### Neutralising antibodies in patients with multiple myeloma receiving maintenance therapy with interferon $\alpha_{2b}$

J.B.G. Bell, R. Barfoot, T. Iveson, R.L. Powles & B.C. Millar

*The McElwain Laboratories, Institute of Cancer Research and the Royal Marsden Hospital, Sutton, Surrey, UK.*

**Summary** In a study of 29 patients who were receiving or had received interferon $\alpha_{2b}$ (IFN-$\alpha_{2b}$) as maintenance therapy for multiple myeloma, antibodies were detected in 58% (17/29) of patients measured by a solid-phase enzyme-linked immunosorbent assay (ELISA). Only 7/17 patients who were positive for antibody in the ELISA had neutralising antibody to IFN-$\alpha_{2b}$, measured by virus growth inhibition. These patients comprised six who were receiving IFN-$\alpha_{2b}$ at the time of assessment and one who had finished treatment. Among patients who were receiving the cytokine, four had progressive disease, one was in complete remission and one in partial remission. Neutralising activity was also detected to natural human leucocyte IFN-$\alpha$ in the same patients. Two patients who were positive for neutralising antibody remain in remission and are continuing to receive IFN-$\alpha_{2b}$. These two patients have since lost their neutralising titre. No neutralising antibody to IFN-$\alpha_{2b}$ or natural human leucocyte IFN-$\alpha$ was detected in serum from six normal donors. The data suggest that neutralising antibody formation in patients with multiple myeloma is not responsible for relapse in patients receiving IFN-$\alpha_{2b}$. The transient nature of neutralising antibody production in patients who remain in remission suggests that this response to IFN-$\alpha_{2b}$ is not associated with memory B cells.

Interferons are a heterogeneous family of proteins which have pleiotropic biological effects in addition to their specific antiviral activity. The availability of highly purified human leucocyte interferon (IFN-$\alpha$) and the development of recombinant products has made it possible to explore the potential therapeutic benefits of these molecules in the treatment of cancer (Mellstedt *et al.*, 1979; Gutterman *et al.*, 1980).

In untreated patients with multiple myeloma, IFN-$\alpha$ used as a single agent has been shown to achieve a response rate of 20-30% (Ahre *et al.*, 1984). Similar response rates have been achieved in patients with advanced multiple myeloma refractory to cytotoxic chemotherapy or in relapse (Costanzi *et al.*, 1985; Rodjer *et al.*, 1990). Addition of IFN-$\alpha$ to conventional induction therapy has increased progression-free survival (IFN-$\alpha_{2b}$: Ludwig *et al.*, 1991; Westin *et al.*, 1991) and response frequency (natural IFN-$\alpha$: Mellstedt *et al.*, 1991; Osterborg *et al.*, 1993) in previously untreated patients. However, in a small non-randomised study there was no significant difference in survival duration between patients given conventional chemotherapy with or without natural leucocyte IFN (Umeda *et al.*, 1991).

Recent studies have concentrated on the use of IFN-$\alpha$s as maintenance therapy for multiple myeloma. Intensification of maintenance therapy with glucocorticoids and IFN-$\alpha_{2b}$ following conventional chemotherapy induced a further reduction of the M component in 50% of patients who responded to chemotherapy (Palumbo *et al.*, 1992). Among 41 patients who failed to achieve remission with chemotherapy, 32% achieved at least a partial response with IFN/dexamethasone, and 42% who had responded to induction showed a further reduction in tumour burden (Salmon *et al.*, 1991). In a non-randomised study, the efficacy of maintenance therapy with recombinant IFN-$\alpha_{2b}$ alone was dependent on the response to intensive therapy with high-dose melphalan (HDM) and total body irradiation. Amongst patients who achieved complete remission (CR) the probability of progression-free survival at 33 months was significantly greater than that for patients who achieved partial response (PR) (Attal *et al.*, 1992). In an ongoing randomised study of 84 patients at the Royal Marsden Hospital, recombinant IFN-$\alpha_{2b}$ increased the median progression-free survival following intensive chemotherapy with HDM and autologous bone marrow rescue (ABMR) (Cunningham *et al.*, 1993). In 62/84 of these patients who achieved CR to HDM/ABMR, IFN-$\alpha_{2b}$ induced a significant prolongation of remission, and 53% of patients remain in stable CR 4 years after intensive therapy. However, among patients who achieved only a partial response or who failed to respond to HDM/ABMR, IFN-$\alpha_{2b}$ failed to prolong progression-free survival. These studies suggest that the efficacy of IFN-$\alpha$s as a maintenance therapy for multiple myeloma is greatest when there has been a significant reduction of the tumour burden.

*In vitro* IFN-$\alpha$ reduced the proliferation of a myeloma cell line at clinically achievable drug concentrations (80-800 U ml$^{-1}$) and acted synergistically with vinblastine and cisplatin (Aapro *et al.*, 1983). However, it is not known whether IFN-$\alpha$s are cytostatic or cytotoxic to myeloma cells *in vivo* or whether they exert their effect(s) directly by inhibiting the proliferation of residual tumour cells or by modulation of the host response. Furthermore, it is not known whether refractiveness to maintenance therapy or relapse in patients receiving IFN-$\alpha$s is associated with the formation of neutralising antibodies to the compound(s).

The development of neutralising antibodies is likely to be dependent on the length of treatment and/or higher doses of IFNs, both of which will contribute to the increased cumulative dose of protein. The route of administration (Konrad *et al.*, 1987), the underlying disease (particularly with regard to the state of immune system) and the source of protein may also contribute to antibody formation. In addition to the development of neutralising antibodies in response to therapy with natural or recombinant molecules, autoantibodies to natural leucocyte IFN have been found in patients with autoimmune disease (Panem *et al.*, 1982), in cancer patients before treatment with IFN-$\alpha$ (Trown *et al.*, 1983) and in sera from normal donors (Ross *et al.*, 1990). In multiple myeloma, which is characterised by immune suppression, some restoration of normal immunoglobulin function occurs following response to treatment, however it is not known whether patients can produce a sustainable response to antigen and consequently whether the development of neutralising antibodies to IFN-$\alpha$ is involved in relapse or refractoriness to further treatment in this disease.

In this report, we set out to determine whether patients with multiple myeloma receiving maintenance therapy with IFN-$\alpha_{2b}$ develop antibodies to the cytokine which are associated with relapse and whether autoantibodies to human leucocyte IFN-$\alpha$ are present in serum.

Correspondence: J.B.G. Bell, The McElwain Laboratories, Institute of Cancer Research, 15 Cotswold Road, Belmont, Sutton, Surrey SM2 5NG, UK.
Received 8 March 1994; and in revised form 16 May 1994.

### Materials and methods

### *Clinical samples*

Patients and donors gave informed consent for blood samples to be taken. Peripheral blood samples were collected by venepuncture from multiple myeloma patients and normal donors at out-patient clinics or as in-patients undergoing treatment. Wherever possible, patients were requested to undergo venepuncture at 3 monthly intervals during treatment with IFN-$\alpha_{2b}$. Serum samples were separated within 2 h of collection. Amongst patients receiving IFN-$\alpha_{2b}$, blood samples were taken within 24 h of drug administration.

Patients with multiple myeloma who presented at the Royal Marsden Hospital, Sutton, received one of two conditioning regimens, either CY-VAMP [i.v. infusion of vincristine, 0.4 mg, and adriamycin, 9 mg $m^{-2}$, over 24 h for 4 days with bolus of methylprednisolone (1.5 g i.v. or orally daily for 5 days) plus cyclophosphamide (500 mg i.v. bolus on days 1, 8 and 15)] or VERCY-VAMP [vincristine, adriamycin, methylprednisolone and cyclophosphamide (doses as before) plus verapamil (10 mg i.v. over 24 h for 5 days)] followed by HDM (either 200 mg $m^{-2}$ with ABMR or 140 mg $m^{-2}$ alone). Patients were given IFN-$\alpha_{2b}$ (INTRON A, Shering-Plough) at $3 \times 10^6$ U $m^{-2}$ subcutaneously three times weekly when the leucocyte count was greater than $3 \times 10^9$ $l^{-1}$ and platelets were greater than $100 \times 10^9$ $l^{-1}$. Among the group of patients, 12 were receiving IFN-$\alpha_{2b}$ as part of the ongoing randomised study. Patients were designated to receive a second course of IFN-$\alpha_{2b}$ if there was a break in therapy greater than 2 weeks not associated with relapse. Eight patients who fulfilled this criterion had had treatment with IFN-$\alpha_{2b}$ interrupted for between 2 weeks and 5 months. One patient who had relapsed during treatment with IFN-$\alpha_{2b}$ was given further intensive therapy followed by IFN-$\alpha_{2b}$ after an interval of 26 months.

### *Clinical status*

A complete remission (CR) was defined as the absence of measurable paraprotein and bone marrow infiltration by myeloma cells of <5%. A partial response (PR) was defined as a paraprotein level reduced by 50% and improvement in all other clinical features sustained for longer than 1 month.

### *Assay for IFN antibodies*

Serum samples were stored at −20°C and were heat inactivated at 56°C for 30 min before being analysed. They were assayed for anti-IFN-$\alpha_2$ antibodies by two different methods.

The presence of binding antibodies to IFN-$\alpha_{2b}$ was measured using a solid-phase enzyme-linked immunoassay (ELISA) (Anawa Labs, Zurich, Switzerland). The assay uses beads coated with recombinant IFN-$\alpha_2$ (rIFN-$\alpha_2$), which were incubated with the serum samples for 24 h at 4°C. The anti-IFN-$\alpha_2$ antibodies in the serum were captured by the beads and, after washing, incubated at 4°C for 24 h with rIFN-$\alpha_2$–peroxidase conjugate. After washing, the enzyme activity on the beads was measured by incubation with enzyme substrate (tetramethylbenzidine) and measurement of the optical density at 450 nm. Human serum with known amounts of rabbit antibody to rIFN-$\alpha_2$ was used as a standard for the calibration curve over the range of 0 to 10 (arbitrary) units $ml^{-1}$. The specificity of the signal obtained with patients' sera was checked with the confirmatory test to exclude false-positive results. In the confirmatory test, the signal of antibody-positive sera was depressed by addition of free rIFN-$\alpha_2$, whereas non-specific binding was not suppressed by the addition of free IFN-$\alpha_2$.

The IFN neutralisation bioassay is based on inhibition by IFN of viral cytopathic effect (Freund *et al.*, 1989; Ross *et al.*, 1990). The rationale for the test is that Cocal virus causes the destruction of cells and the release of infective virion. Addition of IFN-$\alpha$ to cell monolayers before infection with virus inhibits viral replication and conserves the viability of the monolayer. In serum samples which contain antibody to IFN-$\alpha$ neutralisation of IFN-$\alpha$ before addition to HeLa cell monolayers reverses the protective effect of IFN-$\alpha$ and results in destruction of the monolayer by the virus. Cell viability is assessable by measuring the activity of succinate hydrogenase in viable cells with the MTT assay.

### *MTT assay*

3-[4,5-Dimethylthiazol-2-yl]-2,5 diphenyl tetrazolium bromide (MTT Cat. No. M 2128; Sigma, Poole, UK) was dissolved in phosphate-buffered saline (PBS) at a concentration of 5 mg $ml^{-1}$, filtered through a sterilising filter and stored at 4°C in a dark bottle. Lysis solution was 12.5% sodium dodecyl sulphate in 45% dimethyl formamide in water (analytical grade pH 4.7).

A working solution of MTT was prepared for each experiment consisting of one part MTT to eight parts PBS. A 100 µl volume of this solution was added to microtitre wells after removal of the challenge virus (see below); after incubation at 37°C for 2 h, 100 µl of lysis solution was added to dissolve formazan–protein complexes for 45 min. Optical density measurements were made in a Titertek 96-well multiscanner at 540 nm, using an MTT lysis solution as blank.

### *Stock virus*

Confluent monolayers of HeLa cells in $2 \times 175$ $cm^2$ tissue culture flasks were infected with Cocal virus (kindly supplied by W. James, Sir William Dunn School of Pathology, Oxford, UK) in 10 ml of maintenance medium [$\alpha$-modification of Eagle's medium supplemented with 1% fetal calf serum (FCS)] and incubated at 37°C for 24 h. The medium, containing cell debris and virus, was harvested from the cultures, ultrasonicated for 2 min and filtered through a 0.2 µm filter. Cocal was plaque titrated in HeLa cells before use in experiments. Stock virus contained $7 \times 10^7$ plaque-forming units (p.f.u.) $ml^{-1}$. Preliminary experiments were done to optimise the MTT assay. Virus and IFN-$\alpha$ (IFN-$\alpha_{2b}$ and natural human leucocyte IFN-$\alpha$, Cat. no. I 9887, Sigma) titrations were carried out to establish the minimum concentrations of virus that produced the most pronounced change in optical density (OD) and the minimum concentration of IFN-$\alpha$ required to reverse the cytopathic effect at 540 nm. Ninety-six-well microtitre plates were seeded with HeLa cells at a concentration of $10^4$ cells per well in 100 µl of RPMI-1640 medium supplemented with 7.5% FCS. After attachment for 4 h at 37°C, 100 µl of IFN-$\alpha$ was added to give concentrations between 0.05 and 2,500 units $ml^{-1}$ in maintenance medium (see above). Cultures were incubated overnight. Cocal virus was added in 100 µl of maintenance medium at doses of 0.07, 0.70 and 7.0 p.f.u. per cell and incubation continued for 24 h. The medium was discarded into bleach and 100 µl of MTT (1.25 ng $ml^{-1}$) added to each well. After 2 h at 37°C, 100 µl of lysis solution was added to each well and the plate incubated for 45 min before reading at 540 nm in a Titertek multiscanner. The concentration of Cocal virus which gave the optimal change in OD signal was 0.07 p.f.u. per cell. The protective capacity of both human leucocyte IFN-$\alpha$ and IFN-$\alpha_{2b}$ against viral cytopathic effect increased between doses of 0.25 and 5.0 units $ml^{-1}$ IFN-$\alpha$ and thereafter remained constant. All tests with patients' sera were done using IFN-$\alpha$ at a concentration of 5 units $ml^{-1}$ (i.e. 1 unit per well). Polyclonal rabbit anti-human leucocyte IFN-$\alpha$ antibody (r.a-huIFN) was titrated against the optimal concentration of Cocal and 5 units $ml^{-1}$ IFN-$\alpha$ to establish the sensitivity of the MTT assay with respect to changes in OD as a function of antibody concentration (Figure 1). Also, both natural and recombinant IFN-$\alpha$ were titrated against 5 units $ml^{-1}$ r.a-huIFN to determine the sensitivity of the assay with respect to IFN-$\alpha$ concentration (Figure 2). Both human leucocyte IFN-$\alpha$ and IFN-$\alpha_{2b}$ exhibited similar

**Figure 1** Sensitivity of the MTT assay measured at 450 nm demonstrated by titration of polyclonal rabbit anti-human leucocyte IFN-α antibody against Cocal virus and 5 units $ml^{-1}$ IFN-α [both IFN-$\alpha_{2b}$ (Δ) and natural human leucocyte IFN-α (O)] Cell control, HeLa cell monolayer alone; CC + IFN, cell control plus 5 units $ml^{-1}$ IFN-α; virus control, effect of Cocal virus alone on HeLa cell monolayer; VC + IFN, virus plus 5 units $ml^{-1}$ IFN-α.

**Figure 2** Sensitivity of the MTT assay with respect to IFN-α concentration. Titration of both IFN-$\alpha_{2b}$ (Δ) and natural human leucocyte IFN-α (O) against Cocal virus and 5 units $ml^{-1}$ polyclonal rabbit anti-human leucocyte IFN-α antibody measured at 450 nm. Cell and virus controls as in Figure 1.

specificity for commercially available antibody to human leucocyte IFN-α.

*Antibody neutralisation assay*

Sera were diluted into maintenance medium (1:1) containing either 10 units $ml^{-1}$ human leucocyte IFN-α or IFN-$\alpha_{2b}$ and incubated in 96-well tissue culture plates (Nunc) at 37°C for 1 h. A 100 μl volume of the contents of each well was transferred to HeLa cell monolayers in similar plates containing $10^4$ cells per well in 100 μl of growth medium. The final concentration of IFN-α before addition of Cocal was 5 units $ml^{-1}$ (1 unit per well). All subsequent procedures were carried out as described previously. The presence of neutralising antibody to IFN-α in patients' sera was considered significant if the mean OD reading from duplicate samples differed by more than two standard deviations from values at the next successive dilution of serum.

### Results

In the primary screen for antibody to IFN-$\alpha_2$, 48 samples from 29 patients were tested in the solid-phase ELISA assay; 42 samples were taken from patients who were receiving IFN-$\alpha_{2b}$ at the time of testing and six samples from patients who had stopped therapy for at least 8 days. The clinical status of each patient and the isotype of their disease at the time when the first blood sample was taken are shown in Table I. During the period of the study there was a change in clinical status in three patients. Of those patients who were receiving or had received IFN-$\alpha_{2b}$, one patient with IgAκ myeloma progressed from CR to progressive disease (PD), as did another with IgGκ myeloma who had been in PR. Subsequent samples from both patients during PD were included in the test. Additionally, a blood sample from a patient with IgAλ myeloma who was in PR 23 months after intensive therapy was tested 1 month before the start of maintenance IFN-$\alpha_{2b}$ therapy and again during IFN-$\alpha_{2b}$ therapy on progressing to PD.

Nineteen samples from 15 patients who were receiving IFN-$\alpha_{2b}$ at the time of testing were positive for antibody; 11 had PD, two were in PR and two in CR (Table II). Twenty-three samples from 17 patients were negative: nine had PD, six were in PR and two in CR. The median duration of maintenance therapy in both antibody-positive and -negative patients was 12 months (range 5–44 months). Also, there was no significant difference in the distribution of length of treatment with IFN-$\alpha_{2b}$ between antibody-positive and antibody-negative patients.

Among patients who had antibody to IFN-$\alpha_{2b}$, one patient

**Table I** Isotype and clinical status of patients in study

| Isotype | No. of patients | Clinical status[a] CR | PR | PD |
|---|---|---|---|---|
| IgGλ | 6 | | | 6 |
| IgGκ | 14 | 2 | 6 | 6 |
| IgDλ | 1 | | | 1 |
| IgAλ | 3 | 1 | 1 | 1 |
| IgAκ | 1 | 1 | | |
| BJλ | 1 | | 1 | |
| BJκ | 2 | | 1 | 1 |
| Non-secretory | 1 | | | 1 |

[a]CR, complete remission; PR, partial response; PD, progressive disease.

**Table II** Serum samples from multiple myeloma patients tested positive by ELISA for antibodies against interferon-$\alpha_2$

| Patient no. | Isotype | No. of IFN-$\alpha_{2b}$ courses | Duration of IFN-$\alpha_{2b}$ courses (months) | ELISA ($U\ ml^{-1}$) |
|---|---|---|---|---|
| *Myeloma patients receiving IFN-$\alpha_{2b}$ treatment at time of sample* | | | | |
| 1 | IgGλ | 2 | 4+8 | <1.0 |
| 2 | IgGλ | 1 | 26 | 1.3 |
| 3 | IgGκ | 1 | 10 | <1.0 |
| 4 | IgGλ | 1 | 32 | <1.0 |
| 5 | IgGκ | 2 | 1+13 | 7.4 |
| | | 2 | 1+15 | 3.2 |
| 6 | IgAλ | 1 | 12 | 1.0 |
| 7[a] | IgGκ | 1 | 12 | 4.0 |
| | | 2 | 23+10 | 5.5 |
| | | 2 | 23+12 | 8.2 |
| 8 | IgGκ | 2 | 2+14 | 3.4 |
| 9 | IgAκ | 1 | 5 | <1.0 |
| | | 1 | 7 | <1.0 |
| 10[b] | IgGκ | 1 | 31 | <1.0 |
| 11 | IgDλ | 1 | 44 | <1.0 |
| 12[a] | IgGκ | 1 | 44 | 7.6 |
| 13[b] | BJκ | 1 | 12 | 10.1 |
| 14 | Non-secretory | 2 | 5+9 | 8.7 |
| 15 | IgGκ | 2 | 26+11 | 12.5 |
| *Myeloma patients not receiving IFN-$\alpha_{2b}$ treatment at time of sample* | | | | |
| 10[bc] | IgGκ | 1 | 34 | <1.0 |
| | | 1 | 34 | <1.0 |
| 16[d] | IgGλ | 2 | 19 + 5 | <1.0 |

All samples taken from patients in PD except for: [a]patients in CR; [b]patients in PR. [c]Sample taken 1 and 2 months after last dose of IFN-$\alpha_{2b}$. [d]Sample taken 8 days after last dose of IFN-$\alpha_{2b}$.

(no. 7) who remains in CR had detectable antibody during the first course of treatment which continued for 23 months, and the amount of antibody increased during the second course. A second patient (no. 12) who remains in CR has been on maintenance therapy with IFN-$\alpha_{2b}$ for 44 months with a high titre of antibody. Among five patients who had not received IFN-$\alpha_{2b}$ for at least 8 days, antibodies were detected in one patient (no. 16) 8 days after the last dose of IFN-$\alpha_{2b}$ and in a second patient (no. 10) 1 month and 2 months after cessation of treatment. Antibodies had been found in this patient (no. 10) during IFN-$\alpha_{2b}$ maintenance therapy previously (see Table II).

All samples which were positive for antibody in the ELISA were tested for neutralising antibody in the virus growth inhibition assay with both natural human leucocyte IFN-α and IFN$\alpha_{2b}$. Among patients who were receiving IFN-$\alpha_{2b}$, neutralising antibody was detected in seven samples from six patients (Table III). Serum from two of these patients [no. 9 (first sample) and no. 11] were positive for antibodies on the day designated the date of relapse. Despite the presence of neutralising antibody to IFN-$\alpha_{2b}$ in two patients, one was in CR (no. 12) and the other in PR (no. 13). Subsequent samples from these two patients taken after a further 4 months and 8 months (no. 12) or 7 months (no. 13) were negative for neutralising antibodies to both natural IFN-α and IFN-$\alpha_{2b}$. Both patients remain in stable remission. The remaining two patients (nos. 14 and 15) had been maintained on IFN-$\alpha_{2b}$ for 11 months since the diagnosis of PD. One patient (no. 15) had received low doses of melphalan at the onset of PD before starting a second course of IFN-$\alpha_{2b}$. In five samples of serum, the antiviral effect of both natural human leucocyte IFN-α and IFN-$\alpha_{2b}$ was inhibited. In the sixth patient (no. 12), antibody to IFN-$\alpha_{2b}$ was found, but not to natural human leucocyte IFN-α (Table III). In one patient (no. 9) who had activity against both sources of IFN-α, the antibody titre against natural IFN-α was greater than that against IFN-$\alpha_{2b}$, but antibody to IFN-$\alpha_{2b}$ was not detectable in a sample taken 2 months later.

**Table III** Neutralisation of IFN-$\alpha_2$ activity by sera from multiple myeloma patients

| *Patient no.* | *Isotype* | *Neutralising titre against* *IFN-$\alpha_{2b}$* | *Natural IFNα* |
|---|---|---|---|
| 9[d] | IgAκ | 20 | 1,280 |
| | | −ve | 20 |
| 10[bc] | IgGκ | −ve | 20 |
| 11 | IgDλ | 20 | 40 |
| 12[a] | IgGκ | 160 | −ve |
| 13[b] | BJκ | 160 | 20 |
| 14 | Non-secretory | 320 | 320 |
| 15 | IgGκ | 640 | 320 |

All samples taken from patients in PD except for: [a]patient in CR; [b]patients in PR. [c]Sample taken 1 month after last dose of IFN-$\alpha_{2b}$. [d]Samples taken at 5 and 7 months of IFN-$\alpha_{2b}$ treatment.

Among patients who were not receiving IFN-$\alpha_{2b}$, neutralising antibody to natural IFN-α (but not IFN-$\alpha_{2b}$) was detected 1 month after cessation of treatment in one patient (no. 10). In a sample taken 2 months after treatment no antibody was detected to either IFN-α (Table III). This patient has remained in PR.

To determine the nature of neutralising antibody in patients' serum, goat anti-human IgG or IgM was equilibrated with sera at a dilution of 1:20 from all patients who were positive for neutralising antibody before mixing with IFN-$\alpha_{2b}$ or natural IFN-α. Antibody to human IgG removed anti-IFN activity, whereas antibody to human IgM had no effect.

Because other workers have shown that antibodies to natural human leucocyte IFN-α and IFN-$\alpha_{2b}$ are present in normal donor serum (Ross *et al.*, 1990), we examined serum samples from six normal donors and one patient before the start of maintenance IFN therapy. In no instance were neutralising antibodies detectable in our assay system.

In one patient (no. 14) for whom serum samples have been available since relapse and who was still receiving IFN-$\alpha_{2b}$, the neutralising antibody titre to both natural human leucocyte IFN-α and IFN-$\alpha_{2b}$ was 320 after 9 months of maintenance therapy, and this declined to zero as the IFN-$\alpha_{2b}$ course continued and the patient received no other treatment (Table IV).

In a second patient (no. 9) who was in CR and had no detectable antibodies in the ELISA in a sample taken after 3 months' IFN-$\alpha_{2b}$ treatment, antibodies were detected in both assays as therapy continued and the patient entered PD, and then began to decline with time (Table V).

**Table IV** Sequential serum samples from patient 14, who is in PD and receiving IFN-$\alpha_{2b}$, tested for antibodies against IFN-$\alpha_2$

| *Sample no.* | *ELISA for antibodies against IFN-α* | *Neutralising titre against* *IFN-$\alpha_{2b}$* | *Natural IFN-α* | *No. and duration (months) of IFN-$\alpha_{2b}$ courses* |
|---|---|---|---|---|
| I | 8.7 U ml$^{-1}$ | 320 | 320 | 2<br>5+9 |
| II | NA | 20 | 20 | 2<br>5+13 |
| III | NA | −ve | −ve | 2<br>5+16 |
| IV | NA | −ve | −ve | 2<br>5+19 |

NA, not available.

**Table V** Sequential serum samples from patient 9, who was in CR and progressed to PD while receiving IFN-$\alpha_{2b}$, tested for antibodies against IFN-$\alpha_2$

| *Sample no.* | *ELISA for antibodies against IFN-α* | *Neutralising titre against* *IFN-$\alpha_{2b}$* | *Natural IFN-α* | *No. and duration (months) of IFN-$\alpha_{2b}$ courses* |
|---|---|---|---|---|
| I | −ve | NA | NA | 1<br>3 |
| II | <1.0 U ml$^{-1}$ | 20 | 1,280 | 1<br>5 |
| III | <1.0 U ml$^{-1}$ | −ve | 20 | 1<br>7 |

NA, not available.

### Discussion

At the Royal Marsden Hospital the use of IFN-$\alpha_{2b}$ as maintenance therapy in the treatment of multiple myeloma is increasing because of the encouraging results monitored by the prolongation of remission in patients who achieve CR after intensive therapy with HDM/ABMR. Despite the continuing response of more than 50% of these patients for 4 years, IFN-$\alpha_{2b}$ is not curative. Relapse has occurred in approximately 30% of patients who achieved CR, and there appears to be no significant benefit from IFN-$\alpha_{2b}$ maintenance therapy in patients who exhibit PR.

In other haematological disorders, neutralising antibodies have been temporarily associated with a decrease in response to IFN-$\alpha$ (Figlin & Itri, 1988), particularly among patients with B-cell disorders (Leavitt *et al.*, 1987; Quesada *et al.*, 1987; von Wussow *et al.*, 1987; Steis *et al.*, 1988), however neutralising antibodies have had no effect on clinical outcome in some trials (Itri *et al.*, 1987; Steis *et al.*, 1991). In chronic myeloid leukaemia (CML) the development of neutralising antibodies was associated with relapse or refractoriness to IFN-$\alpha_{2b}$ (Freund *et al.*, 1989). In a case report, the evolution of progressive disease in a patient with CML which corresponded to the development of neutralising antibodies to recombinant IFN-$\alpha_{2b}$ was obviated by changing to human leucocyte IFN (Freund *et al.*, 1988).

The data in this study show that, despite impairment of immune function which is associated with multiple myeloma, 62.5% (15/24) of patients who were receiving IFN-$\alpha_{2b}$ at the time of sampling had antibodies to IFN$\alpha_{2b}$ as measured by a solid-phase ELISA. This unexpectedly high number of patients who were antibody positive by ELISA was not confirmed using virus growth inhibition as the end point. Only 7/19 serum samples which were positive by ELISA had neutralising antibody. These samples were from six patients who were receiving IFN-$\alpha_{2b}$, of whom four were in PD, one in PR and one in CR, equivalent to 16% of the total patient population. Despite an increase in tumour mass and paraprotein in the four patients who had PD, immune function was not abolished since the production of antibody involves a B-cell response to antigen. Additionally, although the number of patients with neutralising antibody was small, there was no indication that patients with myeloma of a particular isotype were more prone to antibody formation.

Not only did the ELISA fail to predict the presence of neutralising antibody, but there was no correlation between the magnitude of the antibody response detected by ELISA and the neutralising titre. We suggest that a primary screen using a solid-phase ELISA is of little benefit in predicting neutralising antibody because of the high number of false positives encountered in this study. Other workers have shown that the ability to detect antibody to IFN-$\alpha$ is dependent on the assay system and on the type of recombinant IFN (Figlin & Itri, 1988; Geysen *et al.*, 1988; Steinmann *et al.*, 1992). In a study by Spiegel *et al.* (1986), using an iodinated radioimmunoassay, a low incidence of antibody was found in patients given IFN-$\alpha_{2b}$, suggesting that this molecule had weak antigenicity. However, comparison of radioimmunoassay with an enzyme-linked immunoassay suggested that this was probably due to its lower sensitivity (Itri *et al.*, 1987).

Although neutralising antibody to IFN-$\alpha_{2b}$ was not detected in 1:20 dilution in normal donor serum in this study, the presence of antibody to natural IFN-$\alpha_2$, which cross-reacted with recombinant IFN-$\alpha_{2a}$, IFN-$\alpha_{2b}$ and IFN-$\alpha_{2c}$ in serum at the same dilution from 200 normal donors, prompted Ross *et al.* (1990) to suggest that autoantibodies may be a part of normal immune regulation resulting from exposure to endogenous IFN-$\alpha_2$. Since neutralising antibodies to natural IFN-$\alpha$ were detected in some patients receiving IFN-$\alpha_{2b}$, it cannot be concluded that they resulted from autoantibody production or because of stimulation of pre-B cells. Since the stimulation of memory B cells should result in an increasing neutralising titre as therapy continues, the observation that neutralising antibody was detected transiently in two patients who remain in remission while receiving IFN-$\alpha_{2b}$ suggests that antibody production does not involve memory B cells in these patients. Furthermore, the failure of these antibodies to affect the response in patients in remission suggests that they have a significantly lower affinity for ligand than that of ligand for its cellular receptor.

Although multiple myeloma is associated with the presence of excess plasma cells in the bone marrow, the plasma cell may not represent the major proliferative compartment. The presence of idiotypic B lymphocytes in the peripheral circulation (Ruiz-Arguelles *et al.*, 1984) and the observation that clonogenic myeloma cells have lymphoplasmacytoid as well as plasmacytoid morphology *in vitro* (Millar *et al.*, 1988) suggest that this compartment may consist of progenitor B cells that have undergone gene rearrangement. The apparent failure to evoke a memory B-cell response to IFN-$\alpha_{2b}$ in two patients who remain in remission while receiving the cytokine suggests either that once B cells had encountered antigen they failed to be stored as memory B cells or that once stored they were inhibited from responding to further antigenic stimulus.

Owing to the immune suppressive nature of multiple myeloma, it is unlikely that neutralising antibodies to IFN-$\alpha_{2b}$ will persist as the disease progresses because of the increase in the size of the malignant clone concomitant with a decrease in polyclonal immunoglobulin secretion as a result of inhibition of normal B-cell development. This is likely to occur irrespective of whether memory B cells are involved in the antibody response.

In conclusion, although neutralising antibody to IFN-$\alpha_{2b}$ was detected in approximately 16% of patients with multiple myeloma at relapse, it is unlikely that these antibodies were responsible for the decline in response to IFN-$\alpha_{2b}$, since the appearance of neutralising antibody was also found transiently in patients who remain in remission and are continuing to receive the cytokine.

We thank the Cancer Research Campaign and Medical Research Council for financial support and the nurses and patients of the IBM Unit and Miles Ward at the Royal Marsden Hospital for their cooperation in obtaining the samples.

### References

AAPRO, M.S., ALBERTS, D.S. & SALMON, S.E. (1983). Interactions of human leukocyte interferon with vinca alkaloids and other chemotherapeutic agents against human tumours in clonogenic assays. *Cancer Chemother. Pharmacol.*, **10**, 161-166.

AHRE, A., BJORKHOLM, M., MELLSTEDT, H., BRENNING, G., ENGSTEDT, L., GAHRTON, G., GYLLENHAMMER, H., HOLM, G., JOHANSSON, B., JARNMARK, M., KARNSTROM, L., KILLANDER, A., LERNER, R., LOCKNER, D., LONNQVIST, B., NILSSON, B., SIMONSSON, B., STALFELT, A.M., STRANDER, H., SVEDMYR, E., WADMAN, B. & WEDELIN, C. (1984). Human leucocyte interferon and intermittent high dose melphalan-prednisone administration in the treatment of multiple myeloma: a randomized clinical trial from the Myeloma Group of Sweden. *Cancer Treat. Rep.*, **68**, 1331-1338.

ATTAL, M., HUGUET, F., SCHLAIFER, D., PAYEN, C., LAROCHE, M., FOURNIE, B., MAZIERES, B., PRIS, J. & LAURENT, G. (1992). Intensive combined therapy for previously untreated aggressive myeloma. *Blood*, **79**, 1130-1136.

COSTANZI, J.J., COOPER, M., SCARFFE, J.H., OZER, H., GRUBBS, S.S., FERRARESI, R.W., POLLARD, R.B. & SPIEGEL, R.J. (1985). Phase II study of recombinant alpha 2-interferon in resistant multiple myeloma. *J. Clin. Oncol.*, **3**, 654-659.

CUNNINGHAM, D., POWLES, R., MALPAS, J.S., MILAN, S., MELDRUM, M., VINER, C., MONTES, A., HICKISH, T., NICOLSON, M., JOHNSON, P., MANSI, J., TRELEAVEN, J., RAYMOND, J. & GORE, M.E. (1993). A randomised trial of maintenance therapy with Intron A following high dose melphalan and ABMT in myeloma. *Br. J. Cancer*, **67** (Suppl. XX), 30.

FIGLIN, R.A. & ITRI, L.M. (1988). Anti-interferon antibodies: a perspective. *Semin. Hematol.*, **25** (3) (Suppl. 3), 9–15.

FREUND, M., VON WUSSOW, P., KNUVER-HOPF, J., MOHR, H., POHL, U., EXERIEDE, G., LINK, H., WILKE, H.J. & POLIWODA, H. (1988). Treatment with natural human interferon alpha of a CML-patient with antibodies to recombinant interferon alpha-2b. *Blut*, **57**, 311–315.

FREUND, M., VON WUSSOW, P., DIEDRICH, H., EISERT, R., LINK, H., WILKE, H., BUCHHOLZ, F., LEBLANC, S., FONATSCH, C., DEICHER, H. & POLIWODA, H. (1989). Recombinant human interferon (IFN) alpha-2b in chronic myelogenous leukaemia: dose dependency of response and frequency of neutralizing anti-interferon antibodies. *Br. J. Haematol.*, **72**, 350–356.

GEYSEN, H.M., MASON, T.J. & RODDA, S.J. (1988). Cognitive features of continuous antigenic determinants. *J. Mol. Recognition*, **1**, 32–41.

GUTTERMAN, J.U., BLUMENSCHIEN, G.R., ALEXANIAN, R., YAP, H.Y., BUZDAR, A.V., CABANILLAS, F., HORTOBAGYI, G.N., HERSH, E.M., RASMUSSEN, S.L., HARMON, M., KRAMER, M. & PESTK, S. (1980). Leukocyte interferon-induced tumour regression in human metastatic breast cancer, multiple myeloma and malignant lymphoma. *Ann. Intern. Med.*, **93**, 399–406.

ITRI, L.M., CAMPOIN, M., DENNIN, R.A., PALLERONI, A.V., GUTTERMAN, J.U., GROOPMAN, J.E. & TROWN, P.W. (1987). Incidence and clinical significance of neutralizing antibodies in patients receiving recombinant interferon alfa-2a by intramuscular injection. *Cancer*, **59**, 668–674.

KONRAD, M., CHILDS, A. & MERIGAN, T. (1987). Assessment of the antigenic response in humans to a recombinant mutant interferon beta. *J. Clin. Immunol.*, **7**, 365–375.

LEAVITT, R.D., RATANATHARATHORN, V., OZER, H., ULTMANN, J.E., PORTLOCK, C., MYERS, J.W., KISNER, D., NORRED, S., SPIEGEL, R.J. & BONNEM, E.M. (1987). Alpha-2b interferon in the treatment of Hodgkin's disease and non-Hodgkin's lymphoma. *Semin. Oncol.*, **14** (Suppl. 2), 18–23.

LUDWIG, H., COHEN, A.M., HUBER, H., NACHBAUR, D., JUNGI, W.F., SENN, H., GUNCZLER, P., SCHULLER, J., ECKHARDT, S., SEEWANN, H.L., CAVALLI, F., FRITZ, E. & MICKSCHE, M. (1991). Interferon alfa-2b with VMCP compared to VMCP alone for induction and interferon alfa-2b compared to controls for remission maintenance in multiple myeloma: interim results. *Eur. J. Cancer*, **27** (Suppl. 4), S40–S45.

MELLSTEDT, H., AHRE, A., BJORKHOLM, M., HOLM, G., JOHANSSON, H.B. & STANDER, H. (1979). Interferon therapy in multiple myeloma. *Lancet*, **i**, 245–247.

MELLSTEDT, H., OSTERBORG, A., BJORKHOLM, M., BJOREMAN, M., BRENNING, G., GAHRTON, G., GRIMFORS, G., GYLLENHAMMAR, H., HAST, R., JULLIUSSON, G., JARNMARK, M., KILLANDER, A., KIMBY, E., LERNER, R., MERK, K., OHRLING, M., SIMONSSON, B., SMEDMYR, E., UDEN, A.M., WADMAN, D. & OSBY, E. (1991). Treatment of multiple myeloma with interferon alpha: the Scandinavian experience. *Br. J. Haematol.*, **79** (Suppl. 1), 21–25.

MILLAR, B.C., BELL, J.B.G., LAKHANI, A., AYLIFFE, M.J., SELBY, P.J. & McELWAIN, T.J. (1988). A simple method for culturing myeloma cells from human bone marrow aspirates and peripheral blood *in vitro*. *Br. J. Haematol.*, **69**, 197–203.

OSTERBORG, A., BJORKHOLM, M., BJOREMAN, M., BRENNING, G., CARLSON, K., CELSING, F., GAHRTON, G., GRIMFORS, G., GYLLENHAMMAR, H., HAST, R., JOHANSSON, B., JULIUSSON, G., JARNMARK, M., KIMBY, E., LERNER, R., LINDER, O., MERK, K., NILSSON, B., OHRLING, M., PAUL, C., SIMONSSON, B., SMEDMYR, B., SVEDMYR, E., STALFELT, A.M., STRANDER, H., UDEN, A.M., OSBY, E. & MELLSTEDT, H. (1993). Natural interferon-alpha in combination with melphalan prednisone versus melphalan prednisone in the treatment of multiple myeloma stages II and III: a randomized study from the Myeloma Group of Central Sweden. *Blood*, **81**, 1428–1434.

PALUMBO, A., BOCCADORO, M., GARINO, L.A., GALLONE, G., FRIERI, R. & PILERI, A. (1992). Multiple myeloma: intensified maintenance therapy with recombinant interferon-alpha-2b plus glucocorticoids. *Eur. J. Haematol.*, **49**, 93–97.

PANEM, S., CHECK, I.J., HENRIKSEN, D. & VILCEK, J. (1982). Antibodies to alpha-interferon in a patient with systemic lupus erythematosus. *J. Immunol.*, **129**, 1–3.

QUESADA, J., ITRI, L. & GUTTERMAN, J. (1987). Alpha interferons in hairy-cell leukemia (HCL): a five year follow up in 100 patients. *J. Interferon Res.*, **7**, 678.

RODJER, S., VIKROT, O., WAHLIN, A. & WESTIN, J. (1990). Effect of interferon alpha-2b in advanced multiple myeloma. *J. Intern. Med.*, **227**, 45–48.

ROSS, C., HANSEN, M.B., SCHYBERG, T. & BERG, K. (1990). Autoantibodies to crude human leucocyte interferon (IFN), native human IFN, recombinant human IFN-alpha 2b and human IFN-gamma in healthy blood donors. *Clin. Exp. Immunol.*, **82**, 57–62.

RUIZ-ARGUELLES, G.J., KATZMANN, J.A., GREIPP, P.R., GONCHOROFF, N.J., GARTON, J.P. & KYLE, R.A. (1984). Multiple myeloma: circulating lymphocytes that express plasma cell antigen. *Blood*, **64**, 352–356.

SALMON, S.E., BECKORD, J., PUGH, R.P., BARLOGIE, B. & CROWLEY, J. (1991). Alpha-interferon for remission maintenance: preliminary report on the Southwest Oncology Group Study. *Semin. Oncol.*, **18** (5) (Suppl. 7), 33–36.

SPIEGEL, R.J., SPICEHANDLER, J.R., JACOBS, S.L. & ODEN, E.M. (1986). Low incidence of serum neutralizing factors in patients receiving recombinant alfa-2b interferon (Intron A). *Am. J. Med.*, **80**, 223–228.

STEINMANN, G.G., GOD, B., ROSENKAIMER, F., ADOLF, G., BIDLINGMAIER, G., FRUHBEIS, B., LAMCHE, H., LINDNER, J., PATZELT, E., SCHMAHLING, C. & SCHNEIDER, F.-J. (1992). Low incidence of antibody formation due to long-term interferon-alpha2c treatment of cancer patients. *Clin. Invest.*, **70**, 136–141.

STEIS, R.G., SMITH, J.W., URBA, W.J., CLARK, J.W., ITRI, L.M., EVANS, L.M., SCHOENBERGER, C. & LONGO, D.L. (1988). Resistance to recombinant interferon alpha-2a in hairy-cell leukemia associated with neutralizing anti-interferon antibodies. *N. Engl. J. Med.*, **318**, 1409–1413.

STEIS, R.G., SMITH, J.W., URBA, W.J., VENZON, D.J., LONGO, D.L., BARNEY, R., EVANS, L.M., ITRI, L.M. & EWEL, C.H. (1991). Loss of interferon antibodies during prolonged continuous interferon-alpha 2a therapy in hairy cell leukaemia. *Blood*, **77**, 792–798.

TROWN, P.W., KRAMER, M.J., DENNIN, R.A., CONNELL, E.V., PALLERONI, A.V., QUESADA, J. & GUTTERMAN, J.U. (1983). Antibodies to human leucocyte interferons in cancer patients. *Lancet*, **i**, 81–84.

UMEDA, M., SHIRAI, T., TSUKAHARA, T., KANEKO, H., YAMAUCHI, M., ARIA, N., KOSUGE, T., KATO, M., SHIKOSHI, K. & ANNO, S. (1991). Clinical trial of alpha-interferon (human lymphoblastoid interferon) in combination with VCAP chemotherapy in multiple myeloma. *Rinsho Ketsueki*, **32**, 669–674.

VON WUSSOW, P., FREUND, M., BLOCK, B., DIEDRICH, H., POLIWODA, H. & DEICHER, H. (1987). Clinical significance of anti-interferon-a antibody titres during interferon therapy. *Lancet*, **ii**, 635.

WESTIN, J., CORTELEZZI, A., HJORTH, M., RODJER, S., TURESSON, I. & ZADOR, G. (1991). Interferon therapy during the plateau phase of multiple myeloma: an update of the Swedish study. *Eur. J. Cancer*, **27** (Suppl. 4), S45–S48.